# Hormetic effect of rotenone in primary human fibroblasts

**DOI:** 10.1186/s12979-015-0038-8

**Published:** 2015-09-16

**Authors:** Shiva Marthandan, Steffen Priebe, Marco Groth, Reinhard Guthke, Matthias Platzer, Peter Hemmerich, Stephan Diekmann

**Affiliations:** Leibniz-Institute for Age Research - Fritz Lipmann Institute e.V. (FLI), Beutenbergstrasse 11, D-07745 Jena, Germany; Leibniz Institute for Natural Product Research and Infection Biology - Hans-Knöll-Institute e.V. (HKI), Jena, Germany

## Abstract

**Background:**

Rotenone inhibits the electron transfer from complex I to ubiquinone, in this way interfering with the electron transport chain in mitochondria. This chain of events induces increased levels of intracellular reactive oxygen species, which in turn can contribute to acceleration of telomere shortening and induction of DNA damage, ultimately resulting in aging. In this study, we investigated the effect of rotenone treatment in human fibroblast strains.

**Results:**

For the first time we here describe that rotenone treatment induced a hormetic effect in human fibroblast strains. We identified a number of genes which were commonly differentially regulated due to low dose rotenone treatment in fibroblasts independent of their cell origin. However, these genes were not among the most strongly differentially regulated genes in the fibroblast strains on treatment with rotenone. Thus, if there is a common hormesis regulation, it is superimposed by cell strain specific individual responses. We found the rotenone induced differential regulation of pathways common between the two fibroblast strains, being weaker than the pathways individually regulated in the single fibroblast cell strains. Furthermore, within the common pathways different genes were responsible for this different regulation. Thus, rotenone induced hormesis was related to a weak pathway signal, superimposed by a stronger individual cellular response, a situation as found for the differentially expressed genes.

**Conclusion:**

We found that the concept of hormesis also applies to *in vitro* aging of primary human fibroblasts. However, in depth analysis of the genes as well as the pathways differentially regulated due to rotenone treatment revealed cellular hormesis being related to weak signals which are superimposed by stronger individual cell-internal responses. This would explain that in general hormesis is a small effect. Our data indicate that the observed hormetic phenotype does not result from a specific strong well-defined gene or pathway regulation but from weak common cellular processes induced by low levels of reactive oxygen species. This conclusion also holds when comparing our results with those obtained for *C. elegans* in which the same low dose rotenone level induced a life span extending, thus hormetic effect.

**Electronic supplementary material:**

The online version of this article (doi:10.1186/s12979-015-0038-8) contains supplementary material, which is available to authorized users.

## Introduction

Oxidative stress is defined as an excessive load of Reactive Oxygen Species (ROS) which cause reversible or persistent damage on a cellular or systemic level. However, oxidative stress is dose dependent [1]: high oxygen levels can cause severe damage while low levels of ROS can be beneficial to the organism, resulting in an extended life span [2, 3]. Such biphasic responses to a potentially harmful compound are commonly named hormesis, a concept that was initially postulated by [4] and which was shown to have significant impact on aging with a variety of stressors described [3, 5–10]. Adaptive response processes may explain how increased ROS formation culminates in the promotion of life span [2, 11, 12]. Yet, it is not fully elucidated however, which molecular sensors become directly activated by ROS. In yeast, inhibition of Target of Rapamycin (TOR) extends chronological life span by increasing mitochondrial ROS (mROS) [13]. In *C. elegans*, glucose restriction increases mROS to increase life span [14, 15]. A redox-dependent hormetic response can also regulate the life span of Drosophila [16] and a correlation between increased mROS and prolonged life span was observed in mice [17]. These data can be explained by the hypothesis that a mild increase of ROS and other stressors might lead to a secondary increase in stress defense, culminating in reduced net stress levels and possibly extended life span [14, 18–24]. Currently however, we cannot exclude alternative hypotheses explaining low level ROS induced hormesis. Low mROS levels might also extend life span in humans. *In vivo* data regarding regulation of life span of humans are scarce. Instead, replicative senescence of human cells *in vitro* has been studied as a surrogate for the human life span. In cellular senescence, cells, though metabolically active, stop dividing after a finite number of cell divisions (called the “Hayflick limit”) [25]. Cellular senescence contributes to aging via accumulation of senescent cells in various tissues and organs during life; senescent cells have been hypothesized to disrupt tissue structure and function due to the components they secrete. In primates, the percentage of senescent skin fibroblasts increases with age *in vivo* [26] while senescent cell deletion delays aging-associated disorders in mice [27]. Senescent cells contribute to the decline in tissue integrity and function, rendering the human body susceptible to a number of age-related diseases [28, 29]. These results indicate that cellular senescence is causally implicated in generating age-related phenotypes and that removal of senescent cells can prevent or delay tissue dysfunction and extend health span, linking cellular to tissue and organismal aging. Cellular senescence can be induced by several mechanisms, in most cases involving oxidative or oncogenic stress [30]. Human diploid fibroblasts display an increase in replicative life span under hypoxia [31]. Hypoxia increases cellular ROS levels which were found to be required for the increase of the replicative life span of human fibroblast cells [32]. However, a brief exposure to hyperbaric oxygen or juglone (a compound that generates ROS) can increase life span in *C. elegans* [33]. Rotenone interferes with the electron transport chain in mitochondria, producing increased levels of intracellular ROS due to inhibition of electron transfer from complex I to ubiquinone [34, 35]. Therefore, rotenone can be applied to mimic a physiological increase of ROS as a trigger for cellular aging [36]. Rotenone is a color- and odorless chemical with a broad spectrum of use as an insecticide [37], pesticide [38] and piscicide [39]. Rotenone has been extensively used in age related studies revealing cell line and experimental model specific responses [35, 36, 40–46]. Rotenone induced ROS increase can accelerate telomere shortening and can cause DNA damage, followed by a robust DNA damage response and senescence [47–50]. In addition to aging, mitochondrial dysfunction can result in a number of chronic conditions in humans, including Alzheimer’s disease [51], diabetes [52] and obesity [53]. However, low dose rotenone revealed a lifespan extending capability in *C. elegans* [40].

In this study we investigated the effect of rotenone as a stressor in primary human fibroblasts. We assessed the transcriptomes of primary human fibroblast strains in the presence and absence of mild doses of rotenone during their transition into senescence. We studied the effects of rotenone in MRC-5 fibroblasts derived from male embryonic lung [54], human foreskin fibroblasts (HFF) derived from foreskin of 10 year old donors [55, 56] and WI-38 fibroblasts derived from female embryonic lung [57, 58]. Our data show that the concept of hormesis also applies to *in vitro* aging of primary human fibroblasts.

## Materials and methods

### Cell strains

Primary human fibroblast cell strains were: MRC-5 (*Homo sapiens*, 14 weeks gestation male, from normal lung, normal diploid karyotype, LGC Standards GmbH, Wesel, Germany), WI-38 (*Homo sapiens*, 3 months gestation female, normal lung, normal diploid karyotype, LGC Standards GmbH, Wesel, Germany) and HFF (human foreskin fibroblast, *Homo sapiens*, normal diploid karyotype, a kind gift of T. Stamminger, University of Erlangen, Germany [59]).

### Cell culture

The fibroblast strains were cultured as recommended by LGC in Dulbeccos modified Eagles low glucose medium (DMEM) with L-glutamine (PAA Laboratories, Pasching, Austria), supplemented with 10 % fetal bovine serum (FBS) (PAA). The strains were grown under normal air conditions in a 9.5 % CO_2_ atmosphere at 37 °C. The fibroblasts were maintained separately in the presence of different concentrations (0–2 μM) of rotenone (R8875; Sigma-Aldrich, St. Louis, MO, USA) throughout their span in culture in dim light, due to the light sensitive nature of rotenone [41]. The media were changed and rotenone was supplemented every 3 days to compensate for its short half-life [60].

For sub-culturing, the remaining medium was discarded and cells were washed in 1xPBS (pH 7.4) (PAA) and detached using trypsin/EDTA (PAA). Primary fibroblasts were sub-cultured in a 1:4 (=2 population doublings (PDs)) or 1:2 (=1 PD) ratio. For stock purposes, strains at various PDs were cryo-conserved in cryo-conserving medium (DMEM + 10 % FBS + 5 % DMSO). Cells were immediately frozen at −80 °C and stored for 2–3 days. Thereafter, cells were transferred to liquid nitrogen for long time storage. No re-thawing and re-freezing was done to avoid induction of premature senescence [61].

One vial of each of the 3 different fibroblast strains (MRC-5, HFF and WI-38) were obtained and maintained in culture from an early PD. On obtaining enough stock on confluent growth of the fibroblasts in 75 cm^2^ flasks, cells were sub-cultured into 3 different 75 cm^2^ flasks (“triplicates”) and were maintained until they were senescent in culture.

### Detection of senescence associated β-galactosidase (SA β-Gal)

The SA β-Gal assay was performed as described by [62] in each of the 3 fibroblast strains with and without rotenone. Cells were washed in 1xPBS (pH 7.4) and fixed in 4 % paraformaldehyde (pH 7.4), 10 min at room temperature (RT). After washing the cells in 1xPBS (pH 7.4), staining solution was added consisting of 1 mg/ml X-Gal, 8 mM citric acid/sodium phosphate pH 6.0, 5 mM K_3_Fe(CN)_6_, 5 mM K_4_Fe(CN)_6_, 150 mM NaCl, 2 mM MgCl_2_. The enzymatic reaction occurred without CO_2_ for 4–16 h at 37 °C. After incubation, cells were washed in 1xPBS (pH 7.4) and, in order to visualize cell nuclei, DNA and Senescence Associated Heterochromatin Foci (SAHFs), mounted with 4′-6-diamidine-2-phenyl indole (DAPI) containing Prolong Gold antifade reagent (Invitrogen, Carlsbad, CA, USA). The total number of cells and the number of SA β-Gal stained blue cells were counted. Paired 2-sample type 2 Student’s t-tests, assuming equal variances, were applied to examine the statistical significance of the results obtained by the SA β-Gal assay.

### RNA extraction

Total RNA was isolated using Qiazol (Qiagen, Hilden, Germany) according to the manufacturer’s protocol, with modifications. In brief, the fibroblasts were pelleted in 2 ml safe-lock tubes (Eppendorf, Hamburg, Germany). 1 ml cooled Qiazol and one 5 mm stainless steel bead (Qiagen) were added. Homogenization was performed using a TissueLyzer II (Qiagen) at 20 Hz for 1 min. After incubation for 5 min at RT, 200 ml chloroform was added. The tube was shaken for 15 s and incubated for 3 min at RT. Phase separation was achieved by centrifugation at 12,000 g for 20 min at 4 °C. The aqueous phase was transferred into a fresh cup and 10 mg of glycogen (Invitrogen), 0.16 volume NaOAc (2 M, pH 4.0) and 1.1 volume isopropanol were added, mixed and incubated for 10 min at RT. The RNA was precipitated by centrifugation with 12,000 g at 4 °C for 20 min. The supernatant was removed and the pellet was washed with 80 % ethanol twice and air dried for 10 min. The RNA was re-suspended in 20 ml DEPC-treated water by pipetting up and down, followed by incubation at 65 °C for 5 min. The RNA was quantified with a NanoDrop 1000 (PeqLab, Erlangen, Germany) and stored at −80 °C until use.

### High-throughput RNA sequencing

For quality check, total RNA was analyzed using Agilent Bioanalyzer 2100 (Agilent Technologies, Santa Clara, CA, USA) and RNA 6000 Nano Kit (Agilent) to ensure appropriate RNA quality in terms of degradation. For MRC-5 fibroblasts, the RNA integrity number (RIN) varies between 7.9 and 9.6 with an average of around 8.7. Total RNA was used for Illumina library preparation and next-generation sequencing [63]. Around 2.5 μg total RNA was used for indexed library preparation using Illumina’s TruSeq™ RNA Sample Prep Kit following the manufacturer’s instruction. Libraries were quantified/quality-checked using the Agilent 2100 and DNA 7500 Kit (both Agilent), pooled and sequenced (4 samples per lane) using a HiSeq2000 (Illumina, San Diego, CA, USA) in single-read mode (SR) with 50 cycles using sequencing chemistry v2. Sequencing resulted in approximately 40 million reads with a length of 50 base pairs (bp) per sample. Reads were extracted in FastQ format using CASAVA v1.8.2 (Illumina).

For HFFs, the RIN was roughly 10 for all samples. Library preparation, quantification and quality checking was done as described above, 1 μg total RNA was used as input material. Sequencing was performed in pools of 5 per lane on a HiSeq2500 in high-output mode (50 bp SR, sequencing chemistry v3). Again around 40 million reads were obtained. For extraction of reads in FastQ format, CASAVA v1.8.4 was used.

### RNA-seq data analysis

Raw sequencing data were received in FASTQ format. Read mapping was performed using Tophat 2.0.6 [64] and the human genome references assembly GRCh37.66 (http://feb2012.archive.ensembl.org). The resulting SAM alignment files were processed using featureCounts v1.4.3-p1 [65] and the respective GTF gene annotation, obtained from the Ensembl database [66]. Gene counts were further processed using the R programming language [67] and normalized to Reads per kilo base per million mapped reads (RPKM) values. RPKM values were computed using exon lengths provided by featureCounts and the sum of all mapped reads per sample.

### Sample clustering and analysis of variance

Spearman correlation between all samples was computed in order to examine the variance and the relationship of global gene expression across the samples, using genes with raw counts larger than zero. Additionally, principal component analysis (PCA) was applied using the log2 RPKM values for genes with raw counts larger than zero.

### Detection of differential expression

The Bioconductor packages *DESeq* 1.10.4 [68] and *edgeR* 3.4.2 [69] were used to identify differentially expressed genes. Both packages provide statistics for determining the differential expression in digital gene expression data using a model based on the negative binomial distribution. The non-normalized gene counts have been used here, since both packages include internal normalization procedures. The resulting p-values were adjusted using the Benjamini and Hochberg’s approach for controlling the false discovery rate (FDR) [70]. Genes with an adjusted *p*-value < 0.05, found by both packages on rotenone treatment compared to controls, were assigned as differentially expressed.

### Gene set enrichment analysis to determine the most differentially regulated pathways on aging

We used the R package *gage* [71] in order to find significantly enriched (Kyoto Encyclopedia of Genes and Genomes) KEGG pathways. In case of our RNA-seq data, the calculation, based on the gene counts, was performed as described in the methods manual (http://bioconductor.org/biocLite.R). For the public microarray based datasets, the calculation was based on log2 fold-changes estimated by *limma* (http://bioconductor.org/packages). Estimated p-values were adjusted using the [70] approach for controlling false discovery rates. KEGG pathways were selected as significantly regulated if the FDR corrected p-values were smaller than 0.05. We investigated the most differentially regulated pathways at PDs showing a significant delay in induction of senescence on rotenone treatment, as detected by SA-β Gal.

## Results

We investigated the effect of a low dose of rotenone as a stressor in three different primary human fibroblast cell strains: MRC-5 (male) and WI-38 (female) are from lung tissue while HFFs (male) are skin derived. At several time points in their life span under none or low dose rotenone, we isolated total RNA and assessed the transcriptomes and differentially expressed genes by high-throughput RNA sequencing.

### Impact of rotenone perturbation on induction of senescence and replicative potential of primary human fibroblast strains

**Fig. 1:**
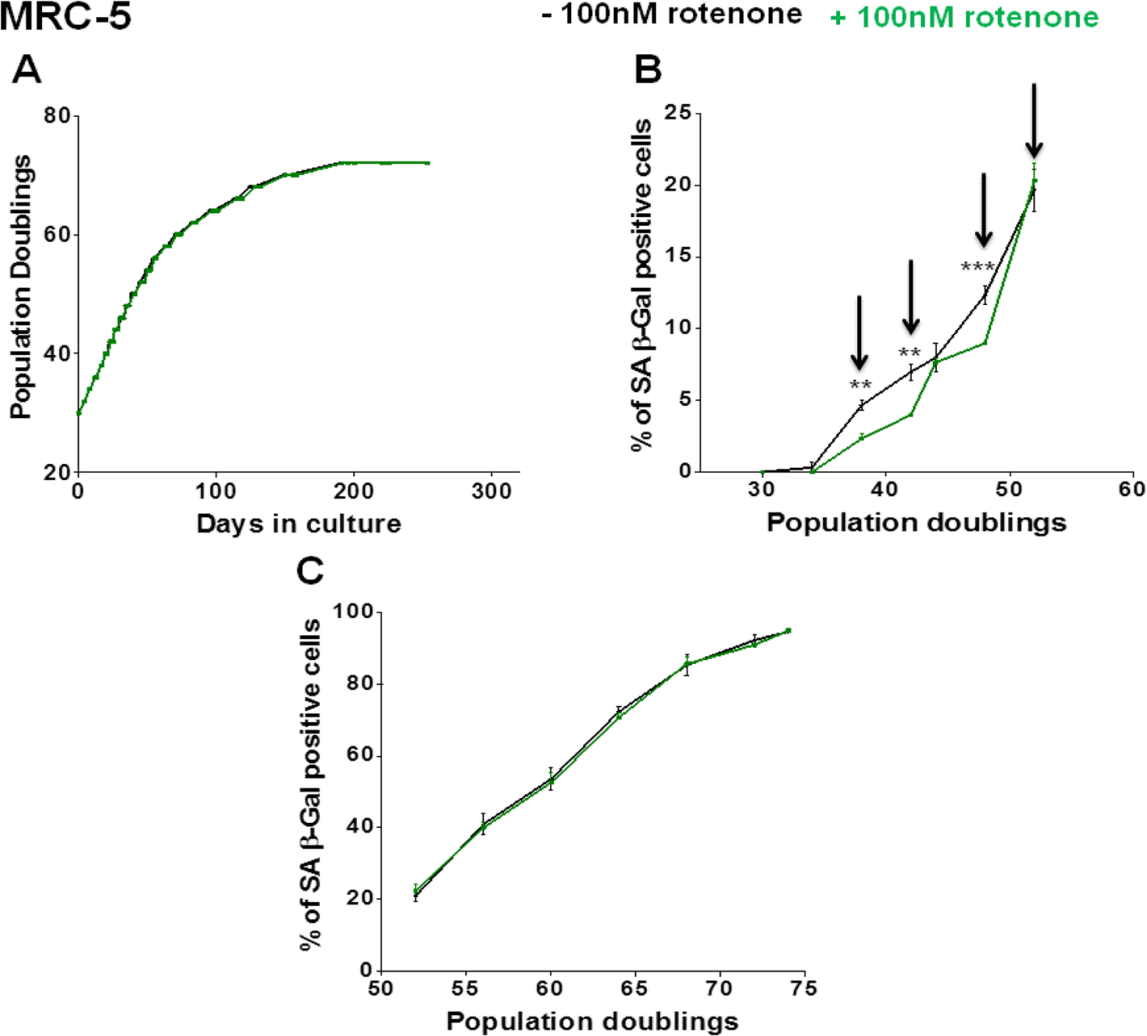
Growth curve and percentage of SA β-Gal positive cells in MRC-5 fibroblasts +/− rotenone treatment. **a** Growth curve of MRC-5 fibroblasts supplemented with 0.1 μM rotenone (*green*) compared to DMSO-treated controls (*black*). **b** Percentage of senescence associated SA β-Gal positive cells in 0.1 μM rotenone-treated young (PD 30) fibroblasts (*green*), compared to DMSO-treated controls (*black*). The arrows indicate the time points at which samples were collected and subjected to next generation sequencing and transcriptome analysis. The bars indicate the mean ± S.D. Values statistically different from their controls (*t*-test) are indicated with an asterix: ** *p* < 0.01, *** *p* < 0.001. **c** Percentage of SA β-Gal positive cells in 0.1 μM rotenone treated mid (PD 52) MRC-5 fibroblasts, compared to untreated controls. *n* = 3 in all cases

**Fig. 2:**
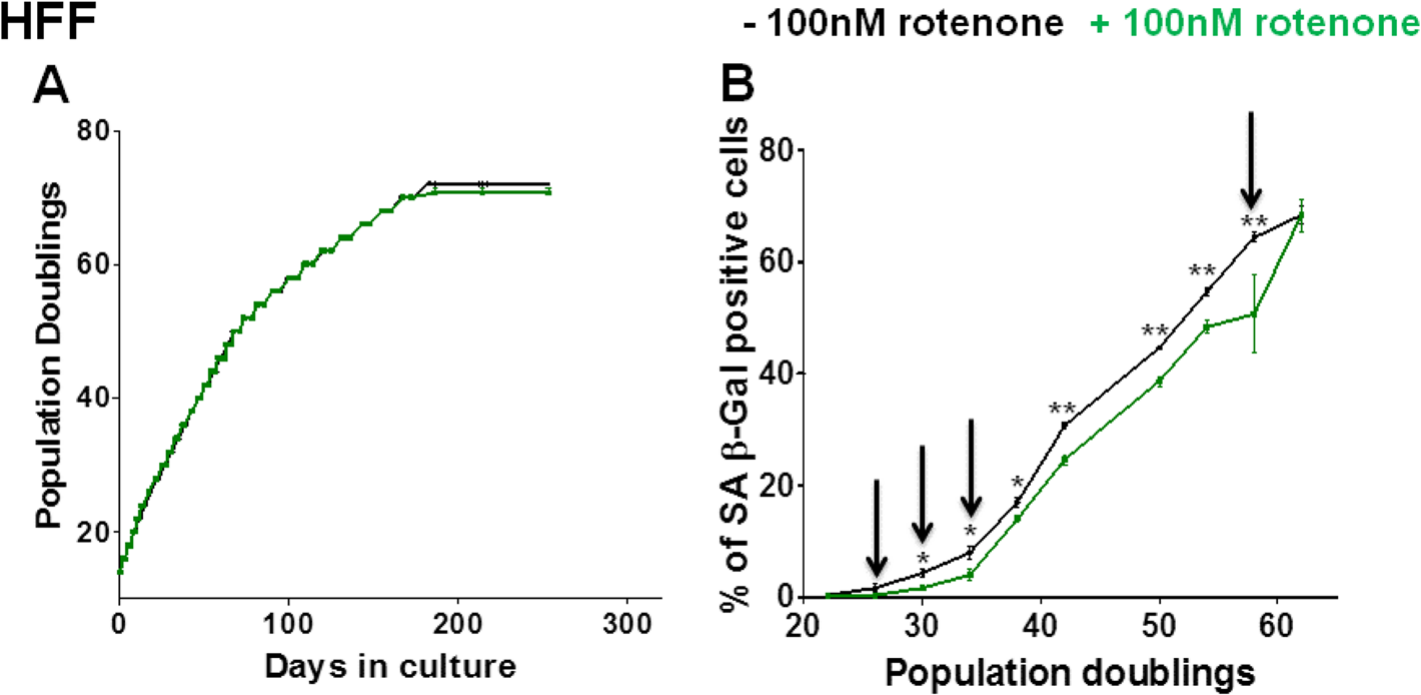
Growth curve and percentage of SA β-Gal positive cells in human foreskin fibroblasts +/− rotenone treatment. **a** Growth curve of HFF fibroblasts supplemented with 0.1 μM rotenone (*green*), compared to DMSO-treated controls (*black*). **b** Percentage of SA β-Gal positive cells in 0.1 μM rotenone treated young HFF fibroblasts (*green*), compared to DMSO-treated controls (*black*). The arrows indicate the time points at which samples were collected and subjected to next generation sequencing and transcriptome analysis. The bars indicate the mean ± S.D. Values statistically different from their controls (*t*-test) are indicated with an asterix: * *p* < 0.05, ** *p* < 0.01. *n* = 3 in all cases

In order to assess a low dose concentration, we added rotenone to the culture medium of growing MRC-5 fibroblasts at various concentrations in the range 0 to 2 μM. Applying rotenone concentrations higher than 0.1 μM induced apoptosis in MRC-5 fibroblasts at different time points during their span in culture (Additional file 1: Table S1), consistent with observations in MCF-7 cells [42]. A concentration of 0.1 μM rotenone was selected as ‘mild stress’ condition since it did not cause any cell death over extended periods of fibroblast passaging (Figs. 1a and 2a; Additional file 2: Figure S1A). In young (PD 30) MRC-5 fibroblasts, 0.1 μM rotenone supplementation resulted in a delay in induction of senescence as indicated by the senescence marker SA β-Gal (Fig. 1b). A similar effect was observed in foreskin fibroblasts (HFF) (Fig. 2b). However, when treating young (PD 32) WI-38 fibroblasts with 0.1 μM rotenone, we detected no delay in senescence induction and no change in the replicative potential (Additional file 2: Figure S1A and B). Taken together, mild oxidative stress treatment using rotenone did not have a life span-extending effect but it induces a delay of senescence, at least in MRC-5 and HFF fibroblasts.

In contrast to young MRC-5, treating older MRC-5 fibroblasts, beginning from the mid stage of their lifespan (PD 50), with 0.1 μM rotenone did not delay senescence induction, compared to DMSO treated controls (Fig. 1c). Thus, low dose rotenone resulted in a delay in senescence induction only when young MRC-5 cells were treated.

### High-throughput RNA sequencing of low dose rotenone treated fibroblasts

Total RNA was isolated from MRC-5 cells at four and from HFF cells at six different time points during their span in culture (Table 1). The samples were subjected to high-throughput RNA sequencing (RNA-seq) [64, 65]. This approach enabled us to quantitatively measure genome-wide polyA^+^ transcript levels and to determine differentially expressed genes (DEGs) in rotenone treated fibroblasts compared to controls. We found that rotenone addition resulted in most DEGs at PDs 42 and 48 in MRC-5 and PDs 26, 30, 34 and 58 in HFFs (Table 1).

**Table 1:** Number of DEGs in primary human fibroblast strains + / - rotenone

Fibroblast	PD+/− rotenone (R)	Number of DEGs
MRC-5	PD38 − R to PD38 + R	1
	PD42 − R to PD42 + R	2086
	PD48 − R to PD48 + R	3527
	PD52 − R to PD52 + R	319
HFF	PD22 − R to PD22 + R	1
	PD26 − R to PD26 + R	4873
	PD30 − R to PD30 + R	8151
	PD34 − R to PD34 + R	6424
	PD58 − R to PD58 + R	5726
	PD74 − R to PD74 + R	60

The number of differentially expressed genes at different population doublings (PDs) in MRC-5 and HFFs supplemented with low dose (0.1 μM) rotenone compared to their corresponding controls (without rotenone but with DMSO). The stringency criteria applied for retrieving the DEGs included *p* < 0.05 and alignment with two statistical packages DESeq [68] and edgeR [69]

### Analysis of variance and sample clustering

**Fig. 3:**
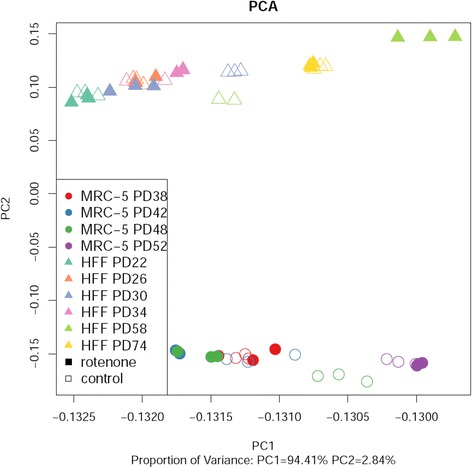
Variance and sample clustering of normalized transcriptome expression values. Principal component analysis (PCA) of MRC-5 (*spheres*) and HFF (*triangles*) cell strains of specific PDs (*indicated by color*) treated with (*filled-in symbols*) and without (*empty symbols*) rotenone. The triplicates are clearly grouped. One of the HFF control sample triplicates of PD34 and PD58 were outliers and were excluded for the analysis, thus only 2 symbols are displayed. The outliers could be attributed to batch effects [116] and their removal from analysis has previously been documented [117]. Interestingly, samples treated with rotenone at low PDs cluster more likely with low PDs which were not treated. Triplicates (*identical symbols*) are clustered indicating small experimental errors. For young (low PDs) and old (high PDs) MRC-5 and HFFs, triplicates with and without rotenone group together indicating little variance due to rotenone treatment. However, for some intermediate PDs, the triplicates with and without rotenone differ strongly indicating transcriptome differences due to rotenone treatment

First, the normalized transcriptome expression values, obtained from high-throughput RNA sequencing, were analyzed using principal component analysis (PCA). PCA reveals the internal structure of the data in a way that best explains their variance. PCA identified the smallest variances between biological replicates (“triplicates”, see Materials & Methods; Fig. 3). PCA indicated a separation of the MRC-5 and HFF cell strains (PC2) as well as the difference between early and late PDs (PC1). The effect of replicative senescence exhibited similarities between MRC-5 and HFF since, for both cell strains, young and old samples are located from the left to the right part in Fig. 3. The strongest effects of the treatment with rotenone, revealed by variances in gene expression, was detected for PD 42 and 48 in MRC-5 and PD 30 and 58 in HFF.

### Rotenone treatment induced differentially expressed genes (DEGs)

Next, we retrieved commonly differentially expressed genes (DEGs) due to rotenone treatment in MRC-5 at PDs 42, 48 and in HFF strains at PDs 26, 30 and 34. These specific PDs were selected due to two criteria: (i) a high number of DEGs retrieved by RNA-seq (Table 1) and (ii) a delay in induction of senescence as measured by SA β-Gal (Figs. 1b and 2b).

In summary, we detected 1113 (568 up- and 545 down-regulated) rotenone treatment induced DEGs in MRC-5 fibroblasts common for both PDs 42 and 48 (*p* < 0.05). In order to identify those genes in this list with the largest expression difference, we implemented the statistical stringency criteria (i) *p* < 0.05, (ii) log2 fold change >1, and (iii) adherence with both statistical packages (DESeq and edgeR). 203 DEGs fulfilled these criteria (160 up- and 43 down-regulated). The most significantly up-regulated genes in this list included *Wnt2*, *CENP-F*, *IGFBP2* and *ALDH1B1*. These four genes had previously been associated with proliferation [72–75]. Due to rotenone treatment significantly down-regulated genes included *Id1*, *Id3*, *MMP10*, *Wnt16* and *CTSK* which have previously been demonstrated to be associated with senescence [76–79]. The significant up-regulation of *IGFBP2*, down-regulation of *Id1* and *Id3* with age and loss of Id function was observed previously for cells transiting into senescence [80–82].

The same approach with three statistical stringency criteria was applied to HFFs. We found a total number of 25 DEGs among the three HFF PDs (18 up- and 7 down-regulated). *Wnt5a* and the cyclin dependent kinase inhibitor (CDKI) *p21*^*CDKN1A*^ transcript levels were significantly up-regulated and *MMP1* expression levels were significantly down-regulated due to 0.1 μM rotenone treatment. Previous studies had associated *Wnt5a* with proliferation [83–85] while the role of *p21* in cell cycle arrest and *MMP1* in senescence is well documented [86, 87].

We then determined the most significant DEGs common among all the above mentioned PDs in both, MRC-5 and HFF, fulfilling the statistical stringency criteria of (i) *p* < 0.05 and (ii) adherence with both statistical packages (DESeq and edgeR) (Additional file 3: Table S2). We speculated that amongst these genes we could identify those genes commonly determining the hormetic effect in both human fibroblast strains. The 12 genes down-regulated due to low dose rotenone treatment included *MMP3* [86, 88, 89] and *CCDC68* while the 18 genes significantly up-regulated in both fibroblast strains included *ENPP2* and the Wnt signaling pathway antagonist *SFRP1* (Additional file 3: Table S2). The role of these genes in senescence has been previously documented [80, 90]. Wnt signaling pathway antagonist *SFRP1*, an inducer of cell cycle arrest [90], was significantly up-regulated in both fibroblasts due to rotenone treatment (see Additional file 3: Table S2) while not being significantly up-regulated during aging in either of the fibroblast strains. However, replicative senescence in HFFs resulted in a significant up-regulation of *SFRP4,* a *SFRP1* family member [90]. Over-expression of *SFRP4* in young (low PD) HFFs resulted in pre-mature senescence induction [80]. However, the observed increase in *SFRP1* expression levels due to rotenone treatment in either of the fibroblast strains did not result in an increase in the percentage of SA β-Gal stained cells (Figs. 1b and 2b).

These genes are significantly differentially regulated in both, MRC-5 and HFF, according to *p* < 0.05 and adherence to the two statistical packages, however, they did not fulfill the criteria of log2 fold change >1. Thus, these common genes were not as significantly differentially regulated due to rotenone treatment as other genes in the single cell strains.

We detected a number of genes regulated in opposite directions when comparing low dose rotenone treatment with cells transiting into senescence. Up-regulation of *CENP-F* and down-regulation of *CTSK* in MRC-5 in response to low dose rotenone was opposite to the differential regulation of these genes in untreated MRC-5 during aging [80]. In HFFs, *MMP1*, a known marker for senescence in fibroblasts [86], was significantly down-regulated due to rotenone treatment. However, these three genes are not commonly regulated in both strains (thus not included in Additional file 3: Table S2). Instead, DEGs commonly most significantly regulated among all the above mentioned PDs in both fibroblast strains included *MMP3* and *CCDC68,* the transcript levels of which were down-regulated in rotenone treated cells but significantly up-regulated with age in replicative senescent MRC-5 fibroblasts [80]. *ENPP2* was significantly up-regulated across all the PDs in both the fibroblast cell strains under mild rotenone stress but significantly down-regulated in aging HFF, however not in MRC-5 fibroblast strains [80]. Thus, we identified four genes (*SFRP1*, *MMP3*, *CCDC68* and *ENPP2*) the expression of which was regulated such that they are potential candidates for hormesis induction in MRC-5 and HFFs. However, in each of the two cell strains, these genes were not the most strongly differentially regulated genes during rotenone treatment, and furthermore, none of the four genes belongs to the pathways which were most differentially regulated due to low dose rotenone treatment in either of the cell lines (see below). In summary, low dose rotenone induced strong differential regulation of a number of genes in both single cell strains, however, the change of expression of genes commonly differentially regulated in both cell strains, which would include the potential hormesis regulators, was weaker. Thus, if there is a common hormesis regulation, it is superimposed by cell strain specific individual responses, with the extreme case of WI-38 cells which do not show hormesis at all.

### Rotenone treatment induced differentially expressed pathways

Using the DAVID functional annotation bioinformatics tool we then asked whether the genes differentially regulated on rotenone treatment in either of the fibroblast strains belonged to any functional category [91]. Genes significantly (*p* < 0.05) down-regulated in MRC-5 or HFF fibroblast strains due to 0.1 μM rotenone treatment were found to be clustered in a group associated with glycoproteins and glycosylation site *O*-linked N-acetylglucosamine (GlcNAc). Previous studies revealed a down-regulation of *O*-GlcNAc mediated glycosylation activity in association with bladder inflammation in mice [92].

Next, using Generally Applicable Gene set Enrichment for pathway analysis (GAGE), we retrieved the KEGG pathways [71] significantly differentially regulated in the MRC-5 and HFF fibroblast strains due to 0.1 μM rotenone treatment (*p*-value < 0.05).

The pathways significantly (*p* < 0.05) up-regulated due to 0.1 μM rotenone treatment in either PD 42 or PD 48 MRC-5 fibroblasts included “DNA replication”, “Cell cycle”, “Oocyte meiosis”, “RNA transport”, “Adherens junction”, “Homologous recombination”, “Mismatch repair”, “Spliceosome”, “Steroid Biosynthesis”, “Nucleotide excision repair”, “Base excision repair”, “Pyrimidine metabolism”, “RNA degradation”, “RNA polymerase” and “Ribosome”. However, only two pathways were commonly up-regulated in MRC-5 for both PDs: “Fatty acid metabolism” and “Propanoate metabolism”. Interestingly, these pathways were significantly down-regulated with age during the transition into senescence in replicatively aged MRC-5 fibroblasts [80]. Eight pathways (“Other glycan degradation”, “Focal adhesion”, “Regulation of actin cytoskeleton”, “Bacterial invasion of epithelial cells”, “Endocytosis”, “ErbB signaling”, “Lysosome” and “Protein processing in endoplasmic reticulum”) were significantly (*p* < 0.05) down-regulated due to rotenone treatment in MRC-5 in at least one of the two PDs (42 and 48). Interestingly, these pathways were significantly up-regulated with age during replicative senescence in MRC-5 fibroblasts [80]. Two pathways, “Lysosome” and “Protein processing in endoplasmic reticulum”, were down-regulated at both PDs.

Twenty-five pathways were found up-regulated (*p* < 0.05) due to low dose rotenone treatment in HFFs at one of the PDs 26, 30 or 34 (Additional file 4: Table S3). Among these, “Ribosome” was the most significantly (*p* < 0.001) up-regulated pathway. As observed for MRC-5 cells, these pathways were down-regulated during replicative HFF senescence [80]. “Ribosome”, “Chemokine signaling pathway” and “NOD-like receptor signaling pathway” was commonly up-regulated in all the three PDs. 30 pathways were found significantly down-regulated in at least one of the three PDs (26, 30 and 34) (Additional file 5: Table S4). The most significantly (*p* < 0.001) down-regulated pathways among these 30 included “Lysosome”, “ABC transporters”, “Drug metabolism-cytochrome P450”, “Metabolism of xenobiotics by cytochrome P450” and “Phagosome”. Interestingly, these pathways were significantly up-regulated with age during replicative senescence in HFFs [80]. Among the 30 pathways, only “Glycosphingolipid biosynthesis - ganglio series” and “Basal cell carcinoma” were down-regulated at all 3 PDs in HFFs.

As the next step, we determined those pathways which were commonly differentially regulated due to rotenone treatment not only for the relevant PDs of either one cell strain (see above) but now also for both cell strains, applying selection criteria of *p* < 0.05. These either up- or down-regulated pathways are listed in Additional file 6: Table S5. A number of pathways, being significantly down-regulated during transition into senescence [80], were up-regulated due to low dose rotenone treatment in both MRC-5 and HFF cell strains (see Additional file 6: Table S5). These pathways included processes associated with cell cycle and DNA repair. The rotenone-induced up-regulation of DNA repair pathways in MRC-5 and HFF fibroblast strains explains the ability of rotenone to act against (oxidative) DNA damage. During replicative senescence, in contrast to rotenone treatment, DNA repair pathways are down-regulated with age so that DNA damage accumulates [58, 80]. The up-regulation of DNA repair genes is consistent with the hormetic effect of rotenone. In addition, we found mRNA splicing genes up-regulated due to rotenone in both fibroblast cell strains (“Spliceosome” pathway). The pathways significantly down-regulated in both fibroblast strains due to rotenone treatment included the “Lysosome” pathway which was significantly up-regulated with age in several fibroblast cell strains of different origin [80]. The up-regulation of the “Lysosome” pathway might reveal the need for degradation of cellular disposals in aging non-replicating cells [93]. “Phagosome” and “ABC transporter” pathways were significantly down-regulated on rotenone treatment in HFF strains while being significantly up-regulated on replicative aging [80]. The up-regulation with age of the “Phagosome” and “ABC transporter” pathways was detected also in other cell systems [94, 95]. In both fibroblast strains, the “Ribosome” pathway was significantly up-regulated due to rotenone treatment and down-regulated during aging. This pathway was significantly up-regulated during brain aging of the short lived fish *N. furzeri* [96] and activated on response to ultraviolet B radiation induced stress [97].

Analyzing the expression levels of the single genes belonging to the significantly differentially regulated pathways on rotenone treatment in both MRC-5 and HFFs, resulted in genes which were only differentially regulated by a log2 fold change <1 compared to untreated controls*.* Furthermore, different genes amongst the pathway members were responsible for the rotenone induced differential regulation of a given pathway. For example in MRC-5 fibroblasts, genes *PGR* and *ADH1B* belonging to pathways “Oocyte meiosis” and “Propanoate metabolism” were the only genes which were up-regulated and *Wnt16* belonging to “Basal cell carcinoma” pathway was the only gene which was down-regulated with a log2 fold change >1. Thus, only in these three cases did we observe a differential up- or down-regulation by log2 fold change >1 compared to controls. All the other genes had a differential expression of a log2 fold change <1. However, in none of these cases such a differential regulation was found for HFF cell strains. The only gene differentially regulated with a log2 fold change >1 in HFF was *CCNB3* belonging to the “Cell cycle pathway”. In summary, the differential regulation of the common pathways was weaker than other pathways identified in the single fibroblast cell strains, and furthermore, within the common pathways different genes were responsible for this different regulation. These findings indicate that hormesis induction due to rotenone treatment is related to a weak pathway signal, superimposed by a stronger individual cellular response, a conclusion as deduced from the DEG results.

We further investigated the expression of genes of the mTOR pathway, considering them being major regulators of cell cycle. However, except for *DDIT4*, belonging to a group of genes responsible for mTORC1 inhibition, all other genes of this pathway were not significantly differentially regulated due to rotenone treatment. Low dose rotenone reduced *DDIT4* expression to a significant extent in both fibroblast strains.

## Discussion

Oxidants are important intracellular signaling molecules, with mROS levels notifying the cell of a changing extracellular environment. Redox-dependent signals induce transcriptional changes in the nucleus leading to cellular decisions including differentiation, growth, cell death and senescence [98, 99]. A particular stressor that is incompatible with cell viability might induce larger quantities of mROS, which non-specifically produce cell damage and subsequent cell death, while another moderate stressor might induce smaller quantities of mROS. Relatively minor damage, induced by intracellular stresses including metabolic perturbations and genomic instability, increases ROS levels, predominantly (although not exclusively) from the mitochondria. Low mROS levels promote adaptation to the stressor and consequently promote cell survival [2, 9] since ROS are not simply a chemical inducing damage but also induce signaling pathways. Thus, the release of oxidants from the mitochondria, or other sources, can provoke a secondary protective response [3, 100]. This phenomenon, termed hormesis (or mitohormesis), posits that low ROS levels can induce cellular defense mechanisms, resulting in health span-promoting effects, while higher ROS levels can cause cellular and systemic damage, culminating in increased mortality [101]. Thus, ROS production and subsequent induction of ROS defense can be essential contributors to longevity.

Here, we induced increasing cellular ROS levels by addition of an external stressor and detected a hormetic effect in human cell strains. We investigated the effect of a range of concentrations of rotenone on the growth of primary human fibroblast strains from different tissue origin (MRC-5, WI-38 and HFF) maintained in culture in triplicates. Supplementing with 0.1 μM rotenone revealed a delay in senescence induction in MRC-5 and HFF (male from different tissue; Figs. 1b and 2b) but not in WI-38 fibroblast strains (female from same tissue as MRC-5; Additional file 2: Figure S1B). This rotenone concentration did not or only to a minor extent affects the cumulative PDs in these three fibroblast strains. To a great degree, cells are reported to keep their tissue-specific phenotype in culture [102]. Interestingly, here we found a similar response for two cell strains (MRC-5 and HFF) from different tissue but major differences between MRC-5 and WI-38 strains, both derived from human lung (though from different genders). A difference between these two cell strains in response to mild stress had been observed by us before: an increase in oxygen levels from 3 % to 20 % induced senescence and a shorter life span in MRC-5 but not in WI-38 cell strains [58], WI-38 cells are thus less sensitive to higher external oxygen levels. Here, in response to rotenone treatment, we confirmed the different properties of these two cell strains. An individual variation in the hormetic response was also observed in the resistance to type 2 diabetes mellitus in humans [103].

Concentrations of rotenone higher than 0.1 μM resulted in apoptosis of the fibroblast strains. Thus, low dose rotenone induced a hormetic effect [104]. The hormetic effect was evident however only in young (low PD) but not in older (higher PDs) cells (Fig. 1c). Possibly, at mid and high PDs, the amount of ROS in the fibroblasts has already increased with age to a value above the hormetic level. The increase of ROS in fibroblasts with age may result in the impairment of mitochondrial membrane potential [105]. In addition, MRC-5 fibroblasts at PD 50 already showed accelerated levels of other typical mediators of senescence including p16, p21, and γH2AX, while these markers are not expressed in MRC-5 at PD 30 [58, 106, 107]. Thus, at higher PDs (PD > 50), the senescence-induced feed-back loop of ROS generation may override any potential hormetic effect of rotenone [108].

The effect of rotenone treatment has previously been investigated in other cell lines and experimental model systems. In MCF-7 cells, 0–20 μM rotenone induced apoptosis in a dose dependent manner [42], consistent with our findings. Seven days of treatment with 0.2 μM rotenone induced senescence in fibroblasts from skin biopsies derived from healthy humans [36] while, similar to our results, the higher concentration of 1 μM after 3 days of treatment resulted in apoptosis [36, 41]. While 0.1 μM rotenone delayed senescence in young PD MRC-5 fibroblasts in our study, the same concentration resulted in depolarization of the mitochondrial membrane potential in skin fibroblasts derived from healthy humans [43]. In muscle derived C2C12 cells, treatment with 0.005 μM rotenone for 48 h was able to induce lipotoxicity [35]. However, 0.2 μM and 0.4 μM rotenone treatment were the highest tolerable concentrations for mtDNA mutations in HCT116 cells and immortalized mouse embryonic fibroblasts, respectively [44]. In rat skeletal and heart mitochondria, 10 μM rotenone treatments significantly increased H_2_O_2_ production [45]. Investigation of rotenone as a stressor in *C. elegans* revealed a dose dependent effect on cell survival. 5 μM rotenone resulted in death of the organism [46], whereas 0.1 μM rotenone resulted in an extended lifespan and improved stress resistance in *C. elegans* [40], effects similar to those observed here for MRC-5 and HFF fibroblasts.

Low level rotenone treatment induced an individual strain specific cellular response. WI-38 cells, found before to be oxygen insensitive [58], did not show a hormetic effect at all while, to a considerable extent, MRC-5 and HFF displayed cell strain specific most differentially expressed genes and a delayed transition into senescence. By statistical selection we determined the most differentially expressed genes common for both strains (Additional file 3: Table S2). Among these, we identified four genes (*SFRP1*, *MMP3*, *CCDC68* and *ENPP2*) with an expression regulation identifying them as potential candidates for hormesis induction in fibroblasts. Over- and under expression of these genes are envisaged to provide experimental proof for this hypothesis.

Several pathways regulated in different directions due to rotenone treatment compared to transition into senescence were identified. Improved DNA repair capacity and cell cycle progression could well be underlying mechanisms inducing a hormetic effect after low dose rotenone treatment. However, on the DEG as well as on the pathway level, the differential regulation of common genes and pathways were weak compared to that of others in the single cell strains. Thus, the rotenone induced common cellular response is a weak signal, superimposed by individual cell-internal gene expression changes. This is consistent with hormesis being a small effect in general, with the extreme case of WI-38 cells not showing a rotenone induced hormesis at all. This suggests that the observed hormetic phenotype does not result from a specific strong gene or pathway regulation but from weak common cellular processes, probably induced by low dose ROS levels [3, 101].

A recent microarray study investigated the effect of 0.6 μM rotenone on fibroblasts from skin biopsies derived from healthy young (23–25 year old) and aged (90–91 year old) human subjects [109], detecting no significantly differentially regulated pathways. This higher rotenone concentration induced apoptosis in the cells studied here. We observed a hormetic effect only in young (low PD) fibroblasts.

0.1 μM rotenone supplementation induced a life span extension in *C. elegans* [40]. As a consequence of the same low dose rotenone treatment, we observed a hormetic effect in two human fibroblast cell strains similar to effects in *C. elegans*. We therefore searched for similarities between the significantly differentially regulated pathways on rotenone treatment in *C. elegans* and the fibroblast cell strains analyzed here. As in our study, rotenone was supplemented throughout the *C. elegans* life span. High-throughput RNA sequencing was conducted at four time points of the *C. elegans* life span (after 1, 5, 10 and 20 days), revealing a number of differentially expressed genes (3460, 158, 2 and 18, respectively) compared to untreated *C. elegans* worms. From our comparison, we excluded the *C. elegans* rotenone data for day one since this may be the immediate organismal response to the addition of a foreign stressor [110, 111]. The comparison of the common most differentially regulated pathways (*p* < 0.05) due to 0.1 μM rotenone treatment in *C. elegans* and human MRC-5 and HFF fibroblasts revealed the common up-regulation of ten pathways (“RNA transport”, “Spliceosome”, “DNA replication”, “Nucleotide excision repair”, “Base excision repair”, “Mismatch repair”, “Homologous recombination”, “Pyrimidine metabolism”, “RNA degradation” and “RNA polymerase”). This might indicate that in both systems low dose rotenone could induce similar mechanisms, resulting in the delay of senescence in fibroblasts and the extension of life span in *C. elegans*. However, none of the genes belonging to the significantly differentially regulated pathways common for both cell strains and *C. elegans* had a log2 fold expression change due to rotenone treatment larger than one in either of the two cell strains. Furthermore, analyzing the genes most differentially expressed due to rotenone treatment in *C. elegans* (on days 5 and 10) revealed no common genes compared to either of the fibroblast strains; the genes most significantly differentially regulated in *C. elegans* have no human orthologues.

Taken together, we find that on the gene and on the pathway level the dominant cellular response to low level rotenone is mostly cell strain specific while the observed common hormetic effect seems to be based on weaker expression differences. This suggests that hormesis is a rather individual response, consistent with [103]. Our results obtained for human fibroblast cell strains show that hormesis occurs already on the cellular level and not necessarily requires high-level, like immune or neuronal, regulatory systems for induction. In animals, immune-system-related and neuronal hormetic effects are common [10, 112, 113] and might add to the hormetic effect induced on the cellular level. Minor stress induced by rotenone or other hormetic agents activates maintenance genes (“vitagenes” [10]), including DNA repair genes as observed here. Our results could be explained by the hypothesis that minor stress induces an over-shooting stress-response that does more than necessary, in this way slightly delaying senescence induction by counteracting aging effects which are due to the time dependent decay of cellular systems. The dose dependent response of hormetic agents has a broad range of biomedical applications [114]. This observed effect *in vitro* if translated *in vivo* might have an impact on longevity in humans.

## Conclusion

In this study, we revealed for the first time a hormetic effect due to 0.1 μM rotenone in MRC-5 and HFF human fibroblast cell strains at early PDs. However, mid and late PD fibroblasts as well as WI-38 cells lacked this effect. Only a limited inhibition of complex I was able to induce hormesis, higher concentrations of rotenone induced apoptosis in the fibroblast cells. Our data suggest that the limited inhibition of complex I, inducing low ROS levels, is beneficial to cell growth while higher levels of complex I inhibition result in adverse effects by promoting diseases [115] and affecting life span. Here we found that on the genes as well as on the pathway level, rotenone induced cellular hormesis is related to weak signals which are superimposed by stronger individual cell-internal responses. This would explain that in general hormesis is a small effect, with WI-38 cells not showing a rotenone induced hormesis at all. Our data indicate that the observed hormetic phenotype does not result from a specific strong well-defined gene or pathway regulation but from weak common cellular processes, induced by low dose ROS levels.

## Data deposition

All reads have been deposited in the NCBI GEO under the accession number GSE64553 and will be made available at the time of publication.

## Additional files

Additional file 1: Table S1. Apoptosis induction by higher concentrations of rotenone treatment in MRC-5 fibroblasts. Numbers of days MRC-5 fibroblasts were maintained in culture before induction of apoptosis (as detected by phosphorylation of p38) in response to different concentrations of rotenone. (DOCX 15 kb) Additional file 2: Figure S1. Growth curve and percentage of SA β-Gal positive cells in WI-38 fibroblasts +/− rotenone treatment. (A) Growth curve of WI-38 fibroblasts supplemented with 0.1 μM rotenone (green), compared to DMSO-treated controls (black). (B) Percentage of SA β-Gal positive cells in 0.1 μM rotenone-treated WI-38 fibroblasts (green), compared to DMSO-treated controls (black). The bars indicate the mean ± S.D. *n* = 3. (DOCX 26 kb) Additional file 3: Table S2. The most significant commonly up- and down-regulated genes in primary human fibroblast strains independent of their cell origin. The common genes most significantly differentially regulated at transcript levels in low dose rotenone treated MRC-5 (PDs 42, 48) and HFFs (PDs 26, 30, 34) compared to untreated controls. These genes were selected according to the statistical stringency criteria of p < 0.05 and adherence with both statistical packages (DESeq and edgeR). (DOCX 20 kb) Additional file 4: Table S3. Up-regulated pathways in human foreskin fibroblasts on low dose rotenone treatment. Pathways significantly (*p* < 0.05) up-regulated on low dose rotenone treatment in one of the three PDs (26, 30 and 34) in HFFs. (DOCX 27 kb) Additional file 5: Table S4. Down-regulated pathways in human foreskin fibroblasts on low dose rotenone treatment. Pathways significantly (*p* < 0.05) down-regulated on low dose rotenone treatment in one of the three PDs (26, 30 and 34) in HFFs. (DOCX 35 kb) Additional file 6: Table S5. Common significantly up- and down-regulated pathways in primary human fibroblast strains independent of their cell origin. Pathways significantly (*p* < 0.05) up- and down-regulated on low dose rotenone treatment in both cell strains (MRC-5 and HFF). (DOCX 26 kb)

## Abbreviations

ROS: Reactive oxygen species
HFF: Human foreskin fibroblasts
DMEM: Dulbeccos modified Eagles low glucose medium
FBS: Fetal bovine serum
CO_2_: Carbondioxide
PD: Population doubling
RT: Room temperature
SAHFs: Senescence associated heterochromatin foci
DAPI: 4′-6-diamidine-2-phenyl indole
PCA: Principle component analysis
RPKM: Reads per kilo base per million mapped reads
FDR: False discovery rate
SA β-Gal: Senescence associated β-Gal
RNA-seq: High-throughput RNA sequencing
CDKI: Cyclin dependent kinase inhibitors
O-GlcNAc: O-linked N-acetylglucosamine
GAGE: Gene set enrichment for pathway analysis
KEGG: Kyoto encyclopedia of genes and genomes

## References

[1] Gems D, Partridge L (2008). Stress-response hormesis and aging: “That which does not kill us makes us stronger”. Cell Metab.

[2] Sena LA, Chandel NS (2012). Physiological roles of mitochondrial reactive oxygen species. Mol Cell.

[3] Yun J, Finkel T (2014). Mitohormesis. Cell Metab.

[4] Southam CM, Ehrlich J (1943). Effects of extract of western red-cedar heartwood on certain wood-decaying fungi in culture. Phytopathology.

[5] Calabrese EJ, Baldwin LA (2002). Defining hormesis. Hum Exp Toxicol.

[6] Cypser JR, Tedesco P, Johnson TE (2006). Hormesis and aging in Caenorhabditis elegans. Exp Gerontol.

[7] Rattan SI (2008). Hormesis in aging. Ageing Res Rev.

[8] Mattson MP (2008). Hormesis defined. Ageing Res Rev.

[9] Ristow M, Schmeisser S (2011). Extending life span by increasing oxidative stress. Free Radic Biol Med.

[10] Calabrese V, Cornelius C, Dinkova-Kostova AT, Iavicoli I, Di Paola R, Koverech A (2012). Cellular stress responses, hermetic phytochemicals and vitagenes in aging and longevity. Biochim Biophys Acta.

[11] Ristow M, Zarse K (2010). How increased oxidative stress promotes longevity and metabolic health: the concept of mitochondrial hormesis (mitohormesis). Exp Gerontol.

[12] Demirovic D, Rattan SI (2013). Establishing cellular stress response profiles as biomarkers of homeodynamics, health and hormesis. Exp Gerontol.

[13] Pan Y, Schroeder EA, Ocampo A, Barrientos A, Shadel GS (2011). Regulation of yeast chronological life span by TORC1 via adaptive mitochondrial ROS signaling. Cell Metab.

[14] Schulz TJ, Zarse K, Voigt A, Urban N, Birringer M, Ristow M (2007). Glucose restriction extends Caenorhabditis elegans life span by inducing mitochondrial respiration and increasing oxidative stress. Cell Metab.

[15] Zarse K, Schmeisser S, Groth M, Priebe S, Beuster G, Kuhlow D (2012). Impaired insulin/ IGF1 signaling extends life span by promoting mitochondrial L-proline catabolism to induce a transient ROS signal. Cell Metab.

[16] Owusu-Ansah E, Song W, Perrimon N (2013). Muscle mitohormesis promotes longevity via systemic repression of insulin signaling. Cell.

[17] Liu X, Jiang N, Hughes B, Bigras E, Shoubridge E, Hekimi S (2005). Evolutionary conservation of the clk-1-dependent mechanism of longevity: loss of mclk1 increases cellular fitness and lifespan in mice. Genes Dev.

[18] Houthoofd K, Braeckman BP, Lenaerts I, Brys K, De Vreese A, Van Eygen S (2002). Axenic growth up-regulates mass-specific metabolic rate, stress resistance, and extends life span in Caenorhabditis elegans. Exp Gerontol.

[19] Johnson TE, Henderson S, Murakami S, de Castro E, de Castro SH, Cypser J (2002). Longevity genes in the nematode Caenorhabditis elegans also mediate increased resistance to stress and prevent disease. J Inherit Metab Dis.

[20] Kharade SV, Mittal N, Das SP, Sinha P, Roy N (2005). Mrg19 depletion increases S. cerevisiae lifespan by augmenting ROS defence. FEBS Lett.

[21] Sinclair DA (2005). Toward a unified theory of caloric restriction and longevity regulation. Mech Ageing Dev.

[22] Vanfleteren JR (1993). Oxidative stress and ageing in Caenorhabditis elegans. Biochem J.

[23] Zarse K, Schulz TJ, Birringer M, Ristow M (2007). Impaired respiration is positively correlated with decreased life span in Caenorhabditis elegans models of Friedreich Ataxia. FASEB J.

[24] Shore DE, Carr CE, Ruvkun G (2012). Induction of cytoprotective pathways is central to the extension of lifespan conferred by multiple longevity pathways. PLoS Genet.

[25] Hayflick L, Moorhead P (1961). The serial cultivation of human diploid cell strains. Exp Cell Res.

[26] Jeyapalan JC, Ferreira M, Sedivy JM, Herbig U (2007). Accumulation of senescent cells in mitotic tissue of aging primates. Mech Ageing Dev.

[27] Baker DJ, Wijshake T, Tchkonia T, LeBrasseur NK, Childs BG, van de Sluis B (2011). Clearance of p16Ink4a-positive senescent cells delays ageing-associated disorders. Nature.

[28] Campisi J (2008). Aging and cancer cell biology. Aging Cell.

[29] Vicencio JM, Galluzzi L, Tajeddine N, Ortiz C, Criollo A, Tasdemir E (2008). Senescence, apoptosis or autophagy? When a damaged cell must decide its path – a mini- review. Gerontology.

[30] Rodier F, Campisi J (2011). Four faces of cellular senescence. J Cell Biol.

[31] Packer L, Fuehr K (1977). Low oxygen concentration extends the lifespan of cultured human diploid cells. Nature.

[32] Bell EL, Klimova TA, Eisenbart J, Schumacker PT, Chandel NS (2007). Mitochondrial reactive oxygen species trigger hypoxia-inducible factor-dependent extension of the replicative life span during hypoxia. Mol Cell Biol.

[33] Cypser JR, Johnson TE (2002). Multiple stressors in Caenorhabditis elegans induce stress hormesis and extended longevity. J Gerontol A Biol Sci Med Sci.

[34] Gondal JA, Anderson WM (1985). The molecular morphology of bovine heart mitochondrial NADH-ubiquinone reductase. Native disulfide-linked subunits and rotenone-induced conformational changes. J Biol Chem.

[35] He Q, Wang M, Petucci C, Gardell SJ, Han X (2013). Rotenone induces reductive stress and triacylglycerol deposition in C2C12 cells. Int J Biochem Cell Biol.

[36] Dekker P, Maier AB, van Heemst D, de Koning-Treurniet C, Blom J, Dirks RW (2009). Stress induced responses of human skin fibroblasts in vitro reflect human longevity. Aging Cell.

[37] Dhaouadi A, Monser L, Adhoum N (2010). Removal of rotenone insecticide by adsorption onto chemically modified activated carbons. J Hazard Mater.

[38] Wood DM, Alsahaf H, Streete P, Dargan PI, Jones AL (2005). Fatality after deliberate ingestion of the pesticide rotenone: a case report. Crit Care.

[39] Caboni P, Sherer TB, Zhang N, Taylor G, Na HM, Greenamyre JT (2004). Rotenone, deguelin, their metabolites, and the rat model of Parkinson’s disease. Chem Res Toxicol.

[40] Schmeisser S, Priebe S, Groth M, Monajembashi S, Hemmerich P, Guthke R (2013). Neuronal ROS signaling rather than AMPK/sirtuin-mediated energy sensing links dietary restriction to lifespan extension. Mol Metab.

[41] Dekker P, de Lange MJ, Dirks RW, Heemst DV, Tanke HJ, Westendorp RG (2011). Relation between maximum replicative capacity and oxidative stress-induced responses in human skin fibroblasts in vivo. J Gerontol A Biol Sci Med Sci.

[42] Deng YT, Huang HC, Lin JK (2010). Rotenone induces apoptosis in MCF-7 human breast cancer cell-mediated ROS through JNK and p38 signaling. Mol Carcinog.

[43] Koopman WJ, Nijtmans LG, Dieteren CE, Roestenberg P, Valsecchi F, Smeitink JA (2010). Mammalian mitochondrial complex I: biogenesis, regulation and reactive oxygen species generation. Antioxid Redox Signal.

[44] Shokolenko I, Venediktova N, Bochkareva A, Wilson GL, Alexeyev MF (2009). Oxidative stress induces degradation of mitochondrial DNA. Nucleic Acids Res.

[45] St-Pierre J, Buckingham JA, Roebuck SJ, Brand MD (2002). Topology of superoxide dismutase production from different sites in the mitochondrial electron transport chain. J Biol Chem.

[46] McKay RM, McKay JP, Avery L, Graff JM (2003). C.elegans: a model for exploring the genetics of fat storage. Dev Cell.

[47] von Zglinicki T (2002). Oxidative stress shortens telomeres. Trends Biochem Sci.

[48] Chen Q, Fischer A, Reagan JD, Yan LJ, Ames BN (1995). Oxidative DNA damage and senescence of human diploid fibroblast cells. Proc Natl Acad Sci U S A.

[49] Lu T, Finkel T (2008). Free radicals and senescence. Exp Cell Res.

[50] Rai P, Onder TT, Young JJ, McFaline JL, Pang B, Dedon PC (2008). Continuous elimination of oxidized nucleotides is necessary to prevent rapid onset of cellular senescence. Proc Natl Acad Sci U S A.

[51] Moreira PI, Zhu X, Wang X, Lee HG, Nunomura A, Petersen RB (2010). Mitochondria: a therapeutic target in neurodegeneration. Biochim Biophys Acta.

[52] Wang CH, Wang CC, Wei YH (2010). Mitochondrial dysfunction in insulin insensitivity: implication of mitochondrial role in type 2 diabetes. Ann N Y Acad Sci.

[53] Unger RH (2002). Lipotoxic diseases. Annu Rev Med.

[54] Holliday R (2014). The commitment of human cells to senescence. Interdiscip Top Gerontol.

[55] Tavalai N, Papior P, Rechter S, Leis M, Stamminger T (2006). Evidence for a role of the cellular ND10 protein PML in mediating intrinsic immunity against human cytomegalovirus infections. J Virol.

[56] Münch S, Weidtkamp-Peters S, Klement K, Grigaravicius P, Monajembashi S, Salomoni P (2014). The tumor suppressor PML specifically accumulates at RPA/Rad51-containing DNA damage repair foci but is nonessential for DNA damage induced fibroblast senescence. Mol Cell Biol.

[57] Trlifjová J, Strízová V, Trlifaj L, Budĕsínský Z, Frühbauer Z (1968). A prolonged cultivation of the human diploid cell strain WI-38. J Hyg Epidemiol Microbiol Immunol.

[58] Marthandan S, Priebe S, Hemmerich P, Klement K, Diekmann S (2014). Long-term quiescent fibroblast cells transit into senescence. PLoS One.

[59] Kronschnabl M, Stamminger T (2013). Synergistic induction of intercellular adhesion molecule-1 by the human cytomegalovirus transactivators IE2p86 and pp 71 is mediated via an Sp1-binding site. J Gen Virol.

[60] Cabras P, Caboni P, Cabras M, Agioni A, Russo M (2002). Rotenone residues on olives and in olive oil. J Agric Food Chem.

[61] Honda S, Hjelmeland LM, Handa JT (2001). Oxidative stress-induced single-strand breaks in chromosomal telomeres of human retinal pigment epithelial cells in vitro. Invest Ophthalmol Vis Sci.

[62] Dimri GP, Lee X, Basile G, Acosta M, Scott G, Roskelley C (1995). A biomarker that identifies senescent human cells in culture and in aging skin in vivo. Proc Natl Acad Sci U S A.

[63] Bentley DR, Balasubramanian S, Swerdlow HP, Smith GP, Milton J, Brown CG (2008). Accurate whole human genome sequencing using reversible terminator chemistry. Nature.

[64] Kim D, Pertea G, Trapnell C, Pimentel H, Kelley R, Salzberg SL (2013). TopHat2: accurate alignment of transcriptomes in the presence of insertions, deletions and gene fusions. Genome Biol.

[65] Liao Y, Smyth GK, Shi W (2014). featureCounts: an efficient general purpose program for assigning sequence reads to genomic features. Bioinformatics (Oxford, England).

[66] Flicek P, Amode MR, Barrell D, Beal K, Brent S, Caravalho-Silva D (2012). Ensemble 2012. Nucleic Acids Res.

[67] R Development Core Team (2008). R: A language and environment for statistical computing.

[68] Anders S, Huber W (2010). Differential expression analysis for sequence count data. Genome Biol.

[69] Robinson MD, McCarthy DJ, Smyth GK (2010). edgeR: a Bioconductor package for differential expression analysis of digital gene expression data. Bioinformatics.

[70] Benjamini Y, Hochberg Y (1995). Controlling the false discovery rate: a practical and powerful approach to multiple testing. J R Stat Soc B.

[71] Luo W, Friedman MS, Shedden K, Hankenson KD, Woolf PJ (2009). GAGE: generally applicable gene set enrichment for pathway analysis. BMC Bioinformatics.

[72] Laoukili J, Kooistra MR, Brás A, Kauw J, Kerkhoven RM, Morrison A (2005). FoxM1 is required for execution of the mitotic programme and chromosome stability. Nat Cell Biol.

[73] Shen L, Zhou S, Glowacki J (2009). Effects of age and gender on WNT gene expression in human bone marrow stroma cells. J Cell Biol.

[74] Hedbacker K, Birsoy K, Wysocki RW, Asilmaz E, Ahima RS, Farooqi IS (2010). Antidiabetic effects of IGFBP2, a leptin-regulated gene. Cell Metab.

[75] Chen Y, Orlicky DJ, Matsumoto A, Singh S, Thompson DC, Vasiliou V (2011). Aldehyde dehydrogenase B1 (ALDH1B1) is a potential biomarker for human colon cancer. Biochem Biophys Res Commun.

[76] Binet R, Ythier D, Robles AI, Collado M, Larrieu D, Fonti C, et al. Wnt16b is a new marker of cellular senescence that regulates p53activity and the phosphoinositide 3-Kinase/AKT pathway. Cancer Res. 2009;69(24):9183–91. 10.1158/0008-5472.CAN-09-1016 PMC7439003 19951988

[77] Davalos AR, Coppe JP, Campisi J, Desprez PY (2010). Senescence cells as a source of inflammatory factors for tumor progression. Cancer Metastasis Rev.

[78] Kong Y, Cui H, Zhang H (2010). Smurf2-mediated ubiquitination and degradation of Id1 regulates p16 expression during senescence. Aging Cell.

[79] Cheng XW, Kikuchi R, Ishii H, Yoshikawa D, Hu L, Takahashi R (2013). Circulating cathepsin K as a potential novel biomarker of coronary heart disease. Atherosclerosis.

[80] Marthandan S, Priebe S, Baumgart M, Groth M, Schaer J, Cellerino A. et al. Conserved senescence pathways in primary human fibroblasts. Mechanisms of Ageing and Development. In press (Under correction revision). 10.1371/journal.pone.0154531 PMC4854426 27140416

[81] Ling F, Kang B, Sun XH (2014). Id proteins: small molecules, mighty regulators. Curr Top Dev Biol.

[82] Hara E, Yamaguchi T, Nojima H, Ide T, Campisi J, Okayama H (1994). Id-related genes encoding helix-loop-helix proteins are required for G1 progression and are repressed in senescent human fibroblasts. J Biol Chem.

[83] Huang CL, Liu D, Nakano J, Ishikawa S, Kontani K, Yokomise H (2005). Wnt5a expression is associated with the tumor proliferation and the stromal vascular endothelial growth factor- an expression in non-small-cell lung cancer. J Clin Oncol.

[84] Kremenevskaja N, von Wasielewski R, Rao AS, Schöfl C, Andersson T, Brabant G (2005). Wnt-5a has a tumor suppressor activity in thyroid carcinoma. Oncogene.

[85] Dissanayake SK, Wade M, Johnson CE, O’Connell MP, Leotlela PD, French AD (2007). The Wnt5A/protein kinase C pathway mediates motility in melanoma cells via the inhibition of metastasis suppressors and initiation of an epithelial to mesenchymal transition. J Biol Chem.

[86] Dimri GP, Itahana K, Acosta M, Campisi J (2000). Regulation of a senescence checkpoint response by the E2F1 transcription factor and p14(ARF) tumor suppressor. Mol Cell Biol.

[87] Kuilman TC, Michaloglou C, Mooi WJ, Peeper DS (2010). The essence of senescence. Genes Dev.

[88] Kang MK, Kameta A, Shin KH, Baluda MA, Kim HR, Park NH (2003). Senescence associated genes in normal human oral keratinocytes. Exp Cell Res.

[89] Wajapeyee N, Serra RW, Zhu X, Mahalingam M, Green MR (2008). Oncogenic BRAF induces senescence and apoptosis through pathways mediated by the secreted protein IGFBP7. Cell.

[90] Elzi DJ, Song M, Hakala K, Weintraub ST, Shiio Y (2012). Wnt antagonist SFRP1 functions as a secreted mediator of senescence. Mol Cell Biol.

[91] Wu Y, Zang WD, Jiang W (2008). Functional analysis of differentially expressed genes associated with glaucoma from DNA microarray data. Genet Mol Res.

[92] Chung S, Kang DO, Yamzon J, Warburton D, Koh CJ (2010). O-GlcNAc mediated glycosylation down-regulation in mice with cyclophosphamide induced cystitis. J Urol.

[93] Zealley B, de Grey ADNJ (2011). Commentary on some recent theses relevant to combating aging. Rejuvenation Res.

[94] Feeney-Burns L, Eldred GE (1983). The fate of the phagosome: conversion to ‘age pigment’ and impact in human retinal pigment epithelium. Trans Ophthalmol Soc U K.

[95] Efferth T (2003). Adenosine triphosphate-binding cassette transporter genes in ageing and age-related diseases. Ageing Res Rev.

[96] Baumgart M, Groth M, Priebe S, Savino A, Testa G, Dix A (2014). RNA-seq of the aging brain in the short lived fish N.furzeri – conserved pathways and novel genes associated with neurogenesis. Aging Cell.

[97] Tsai ML, Chang KY, Chiang CS, Shu WY, Weng TC, Chen CR (2009). UVB radiation induced persistent activation of ribosome and oxidative phosphorylation pathways. Radiat Res.

[98] Schieber M, Chandel NS (2014). ROS function in redox signaling and oxidative stress. Curr Biol.

[99] Holmström KM, Finkel T (2014). Cellular mechanisms and physiological consequences of redox dependent signalling. Nat Rev Mol Biol Cell.

[100] Kawagishi H, Finkel T (2014). ROS and disease: finding the right balance. Nat Med.

[101] Ristow M (2014). Mitohormesis explains ROS-induced health benefits. Nat Med.

[102] Kipling D, Jones DL, Smith SK, Giles PJ, Jennert-Burston K, Ibrahim B (2009). A transcriptomic analysis of the EK1.Br strain of human fibroblastoid keratocytes: the effects of growth, quiescence and senescence. Exp Eye Res.

[103] Kolb H, Eizirik DL (2012). Resistance to type 2 diabetes mellitus: a matter of hormesis?. Nat Rev Endocrinol.

[104] Luna-Lopez A, Gonzalez Puertos VY, Lopez-Diazquerrero NE, Königsberg M (2014). New considerations on hormetic response against oxidative stress. J Cell Commun Signal.

[105] Koziel R, Greussing R, Maier AB, Declercq L, Jansen-Durr P (2011). Functional interplay between mitochondrial and proteasome activity in skin aging. J Invest Dermatol.

[106] Schäuble S, Klement K, Marthandan S, Münch S, Heiland I, Schuster S (2012). Quantitative model of cell cycle arrest and cellular senescence in primary human fibroblasts. PLoS One.

[107] Klement K, Melle C, Murzik U, Diekmann S, Norgauer J, Hemmerich P (2012). Accumulation of annexin A5 at the nuclear envelope is a biomarker of cellular aging. Mech Ageing Dev.

[108] Passos JF, Nelson G, Wang C, Richter T, Simillion C, Proctor CJ (2010). Feedback between p21 and reactive oxygen species production is necessary for cell senescence. Mol Syst Biol.

[109] Dekker P, Gunn D, McBryan T, Dirks RW, van Heemst D, Lim FL (2012). Microarray based identification of age-dependent differences in gene expression of human dermal fibroblasts. Mech Ageing Dev.

[110] Rodriguez M, Snoek LB, De Bono M, Kammenga JE (2013). Worms under stress: C.elegans stress response and its relevance to complex human diseases and aging. Trends Genet.

[111] Halliwell B (2014). Cell culture, oxidative stress, and antioxidants: avoiding pitfalls. Biomed J.

[112] Calabrese EJ (2005). Hormetic dose–response relationships in immunology: occurrence, quantitative features of the dose response, mechanistic foundations, and clinical implications. Crit Rev Toxicol.

[113] McClure CD, Zhong W, Hunt VL, Chapman FM, Hill FV, Priest NK (2014). Hormesis results in trade-offs with immunity. Evolution.

[114] Calabrese EJ (2014). Hormesis: from mainstream to therapy. J Cell Commun Signal.

[115] Schapira AH (2010). Complex I: inhibitors, inhibition and neurodegeneration. Exp Neurol.

[116] Leek JT, Scharpf RB, Bravo HC, Simcha D, Langmead B, Johnson WE, et al. Tackling the widespread and critical impact of batch effects in high-throughput data. Nat Rev Genet. 2010;11(10):733–39. 10.1038/nrg2825 PMC3880143 20838408

[117] Kauffmann A, Huber W. Microarray data quality control improves the detection of differentially expressed genes. Genomics. 2010;95(3):138–42. 10.1016/j.ygeno.2010.01.003 20079422

