# Supplementary figures and images for: Hormetic effect of rotenone in primary human fibroblasts
Source: Immun Ageing. 2015 Sep 16;12:11. doi: 10.1186/s12979-015-0038-8 (PMC4572608; doi:10.1186/s12979-015-0038-8)

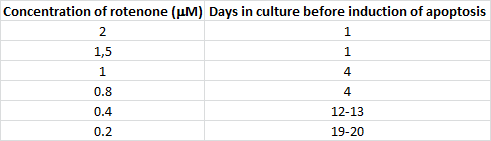

Supplement: Additional file 1: Table S1. — Apoptosis induction by higher concentrations of rotenone treatment in MRC-5 fibroblasts. Numbers of days MRC-5 fibroblasts were maintained in culture before induction of apoptosis (as detected by phosphorylation of p38) in response to different concentrations of rotenone. (DOCX 15 kb) [file 12979_2015_38_MOESM1_ESM.docx]

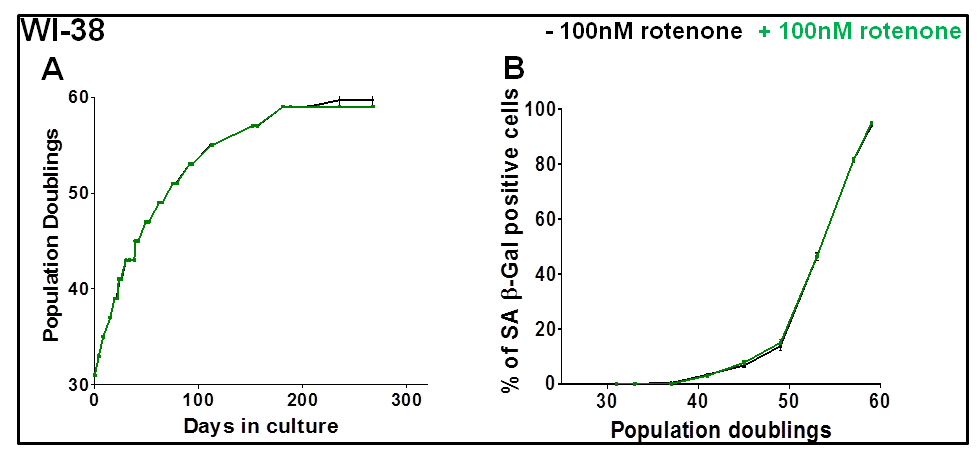

Supplement: Additional file 2: Figure S1. — Growth curve and percentage of SA β-Gal positive cells in WI-38 fibroblasts +/− rotenone treatment. (A) Growth curve of WI-38 fibroblasts supplemented with 0.1 μM rotenone (green), compared to DMSO-treated controls (black). (B) Percentage of SA β-Gal positive cells in 0.1 μM rotenone-treated WI-38 fibroblasts (green), compared to DMSO-treated controls (black). The bars indicate the mean ± S.D. n = 3. (DOCX 26 kb) [file 12979_2015_38_MOESM2_ESM.docx]

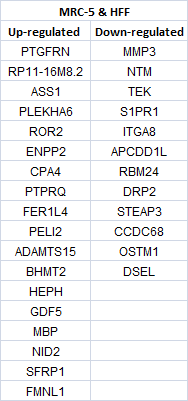

Supplement: Additional file 3: Table S2. — The most significant commonly up- and down-regulated genes in primary human fibroblast strains independent of their cell origin. The common genes most significantly differentially regulated at transcript levels in low dose rotenone treated MRC-5 (PDs 42, 48) and HFFs (PDs 26, 30, 34) compared to untreated controls. These genes were selected according to the statistical stringency criteria of p < 0.05 and adherence with both statistical packages (DESeq and edgeR). (DOCX 20 kb) [file 12979_2015_38_MOESM3_ESM.docx]

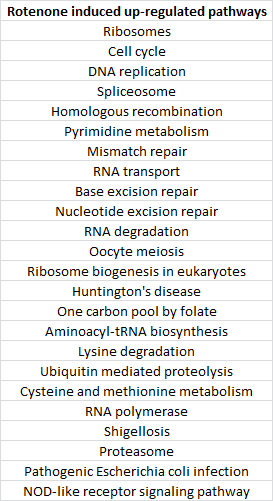

Supplement: Additional file 4: Table S3. — Up-regulated pathways in human foreskin fibroblasts on low dose rotenone treatment. Pathways significantly (p < 0.05) up-regulated on low dose rotenone treatment in one of the three PDs (26, 30 and 34) in HFFs. (DOCX 27 kb) [file 12979_2015_38_MOESM4_ESM.docx]

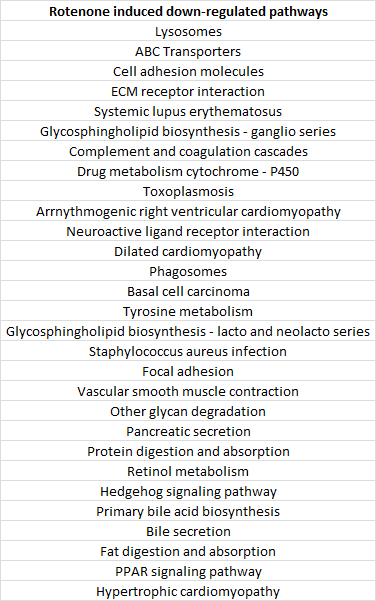

Supplement: Additional file 5: Table S4. — Down-regulated pathways in human foreskin fibroblasts on low dose rotenone treatment. Pathways significantly (p < 0.05) down-regulated on low dose rotenone treatment in one of the three PDs (26, 30 and 34) in HFFs. (DOCX 35 kb) [file 12979_2015_38_MOESM5_ESM.docx]

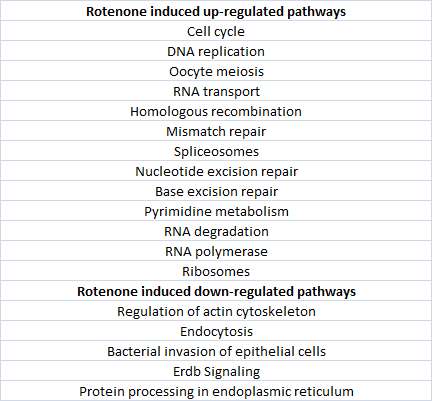

Supplement: Additional file 6: Table S5. — Common significantly up- and down-regulated pathways in primary human fibroblast strains independent of their cell origin. Pathways significantly (p < 0.05) up- and down-regulated on low dose rotenone treatment in both cell strains (MRC-5 and HFF). (DOCX 26 kb) [file 12979_2015_38_MOESM6_ESM.docx]
